# A new complication of retained surgical gauze: development of malignant fibrous histiocytoma – report of a case with a literature review

**DOI:** 10.1186/1477-7819-10-139

**Published:** 2012-07-09

**Authors:** Mehmet Kaplan, Halil İbrahim İyiköşker

**Affiliations:** 1 Department of General Surgery, Medical Park Gaziantep Hospital, Mucahitler mah. 52063 sk. No:2 Sehitkamil, Gaziantep, 27090, Turkey; 2 Department of General Surgery, Dr. Ersin Arslan State Hospital, Gaziantep, Turkey

**Keywords:** Malignant fibrous histiocytoma, Retained surgical gauze, Gossypiboma, Textiloma, Retained foreign body, Soft tissue sarcoma

## Abstract

**Background:**

Primary visceral malignant fibrous histiocytoma (MFH) is a rare disease, and few cases have been reported in the English literature. However, retained foreign bodies in the abdomen after surgical procedures are important causes of intra-abdominal infections. For legal and ethical reasons, there are few publications in the literature. In this article, we describe for the first time a case of malign abdominal fibrous histiocytoma associated with a surgical sponge forgotten in the abdominal cavity a long time ago.

**Case presentation:**

A 64-year-old male presented to our surgical department with cachexia, abdominal pain, distention and pyrexia of unknown origin. He had a medical history of abdominal surgery for peptic ulcer perforation 32 years ago. Clinical examination revealed fever with a distended and painful abdominal wall. Radiological imaging of the abdomen showed multiple heterogeneous masses in one large cystic cavityalmost completely filling the abdomen. The patient underwent a laparotomy, and interestingly, opening the cyst revealed retained surgical gauze (RSG). The origin of the tumor was the visceral peritoneum, and it was excised totally.

**Conclusions:**

Primary intra-abdominal MFH can present as a complication of long-lasting RSG. Therefore, clinicians must remember this while establishing the differential diagnosis for patients with a history of previous abdominal surgery and presenting with symptoms associated with both the tumor and systemic inflammatory response.

## Background

Futoshi Okada began a review article with the statement, “Foreign-body-induced carcinogenesis is a traditional, maybe old, way of understanding cancer development” [1]. He postulated that exogenously incorporated foreign bodies can induce tumors. Fortunately, this phenomenon is uncommon in humans. Only a few reports describe the development of tumors in association with foreign bodies, and in most of them, the tumor is a malignant fibrous histiocytoma (MFH) [2-4].

Concerning foreign bodies, retained surgical gauze (RSG)-induced MFH has been reported in only one case [2]. In this report, the site of the tumor was the thorax, whereas development of MFH in the abdomen, in association with RSG, has never been reported.

We report such a case involving 65-year-old male with previous history of abdominal surgery, who presented with a huge, painful cystic mass and pyrexia of unknown origin. Subsequently, primary intra-abdominal MFH was found as a complication of long-term RSG.

## Case presentation

A 65-year-old-male presented to the surgical outpatient clinic of Medical Park Gaziantep Hospital with abdominal pain and distention, anorexia, weight loss and pyrexia. Abdominal pain was of recent onset and mainly in the central part of abdomen, but he had had a low-grade fever for at least 6 months. His pyrexia was intermittent, and most common at night and early in the morning. He had a medical history of abdominal surgery for peptic ulcer perforation 32 years ago. Clinical examination revealed a firm, vaguely defined, tender mass in the abdomen from the epigastrium to the pelvis. Blood results showed persistently high ESR (>50), high CRP (>200), leukocytosis, mildly raised alkaline phosphatase levels and anemia (normochromic, normocytic). There was no obvious source of infection that could cause the fever. Repeated blood cultures did not yield any bacterial growth. There was no improvement of the pyrexia after treating the patient with broad-spectrum antibiotics.

**Figure 1:**
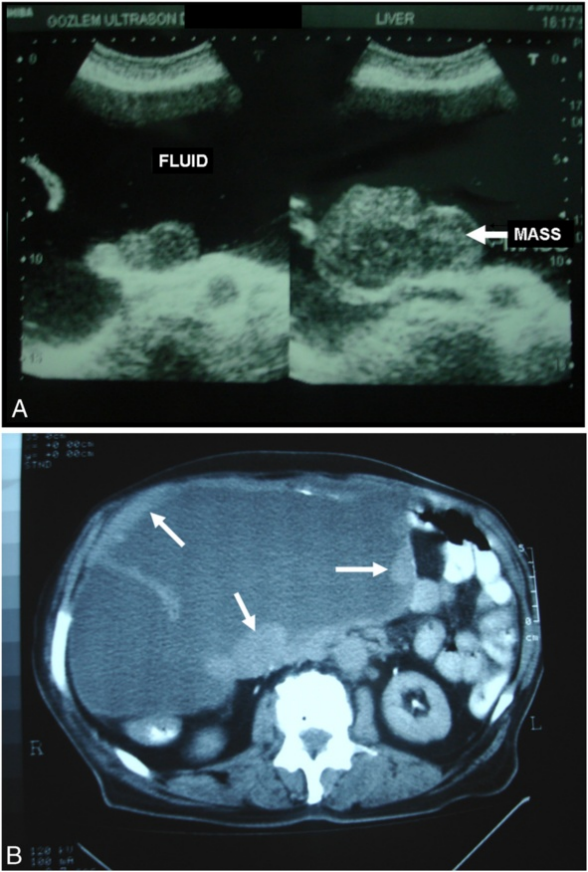
**(A) An abdominal USG revealed multiple heterogeneous masses (*arrows*) in one large cystic cavity.** (**B**) An abdominal CT revealed a huge polycystic tumor, almost completely filling the abdomen, with a part of the thickened wall (*arrows*).

**Figure 2:**
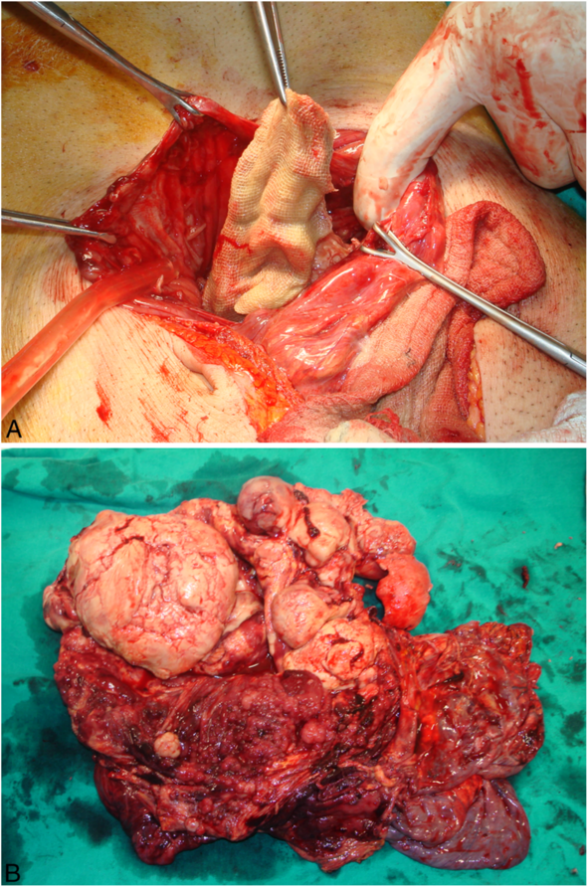
**(A) A laparotomy revealed a huge cystic tumor.** After opening its thickened wall, RSG was found. (**B)** The operative specimen revealed a polycystic tumor with multiple solid components.

Ultrasound and CT scan of the abdomen was performed, which showed multiple heterogeneous masses in one large cystic cavity almost completely filling the abdomen (Figure 1A and B). After the patienthad been consented for surgery, the thick cyst wall was opened, and 3 L of a clear fluid was aspirated, whose subsequent cytological examination determined class I. Surprisingly, a RSG was found at the bottom of the cavity and retrieved immediately (Figure 2A). Then the tumor was excised totally along with visceral peritoneum and mesorectum (Figure 2B). There was no major vascular or adjacent tissue invasion. The mesentery, including the tumor, was well circumscribed by the surrounding organs. Therefore, the origin of this tumor was thought to be the mesentery and visceral peritoneum in conjunction with the foreign body. The liver, spleen and pelvis had no local lesions. The patient had an uneventful postoperative recovery, and the pyrexia resolved completely following surgery.

A histopathological examination revealed proliferation of pleomorphic cells in a storiform pattern. Mitotic figures were also frequently observed. Immunohistochemical analyses indicated that many of the tumor cells were positive for vimentin, while they were negative for cytokeratins, desmin, S-100 protein, actins, c-kit and CD34. These features are compatible with MFH of a storiform-pleomorphic subtype.

Fourteen months following resection of the tumor, the patient was re-admitted with abdominal pain, weight loss and anemia. On CT scanning, he was found to have local recurrence of the tumor as well as liver metastases. At this stage, the patient was referred to the oncology department, but unfortunately, despite treatment, the patient died of progressive disease 2 months later.

## Discussion

An abdominal textiloma, a RSG left after a surgical operation, is a serious medico-legal problem. Clinically, it can lead to abdominal pain, intestinal obstruction, digestive tract fistula or inflammatory tumor formation [5-7]. Sometimes, textiloma is asymptomatic, discovered incidentally during an imaging study done for another reason [7,8]. In most cases, it manifests radiologically as a hyperreflective lesion with a hypoechoic rim and a strong posterior shadow on ultrasound, and a whorl-like spongiform hypodense mass with a thick peripheral rim on CT [8]. The complications and sequelae of the RSG object vary according to its location in the body. It has been reported that, acutely, it can lead to a septic course resulting in abscess or granuloma formation, whereas in delayed presentations, it can lead to adhesion formation, encapsulation, cyst formation, fistulization or direct migration to a lumen, intestinal obstruction, malabsorption and gastrointestinal hemorrhage [5-8], or even a sudden death [9].

It has long been known that foreign bodies incorporated in the human body, both for treatment purposes or accidentally, can induce cancer [1,10]. However, reporting a cancer development as a complication of textiloma is limited to only one case [2]. To our knowledge, this is the second report of malignant transformation at the site of a RSG in a man and the first report of a primary intra-abdominal MFH arising around a textiloma. The latent period from the presence of the foreign body to the appearance of a tumor in humans is extremely long. The estimated average period is 20 years [1]. This is consistent with the present case, as the surgical gauze had been forgotten 32 years ago during abdominal surgery.

It was postulated in the last decade that foreign bodies may induce an inflammation-based carcinogenesis. Some properties, such as the shape, size, porosity, smoothness and hardness of foreign bodies, and the gender of the host, influence the carcinogenic potential. Accordingly, under appropriate conditions, it is possible that the textile material can cause cancer [1,10]. In the current case, although the exact mechanisms are unknown, theoretically it is clear that the RSG induced the development of MFH after a long latent period, probably in an inflammation-based manner.

MFH is a sarcoma of mesenchymal origin affecting soft tissues of the body and is considered the most common soft tissue sarcoma in adults. Its occurrence has been reported in almost all parts of the body, particularly the extremities, trunk and retroperitoneum [2,11,12]. Rarely, it can affect intra-peritoneal organs [13-15]. Great interest and controversy have been generated concerning the pathological and oncological aspects of MFH [11,12] since the first description by O’Brien and Stout [16]. MFH typically manifests as a broad range of histopathological appearances and is currently classified into five subtypes: storiform-pleomorphic, myxoid, giant cell, inflammatory and angiomatoid subtypes [12]. In the current case, the tumor had the storiform-pleomorphic subtype of MFH, which historically comprises the majority of MFH cases, accounting for up to 70% of all reported cases.

In addition to the symptoms, which depend on the primary site of the body affected by the tumor, symptoms of systemic illness caused by the tumor may also be the presenting complaint. Our patient is a good example to support this claim. The fever of unknown origin at the patient’s presentation was probably caused by tumor necrosis and the release of inflammatory and pyrogenic factors in addition to the systemic effect of RSG. Therefore, in a patient who has a history of abdominal surgery and presents with the complaints of abdominal pain, distention and pyrexia of unknown origin, a CT scan should be made early in the examination as it can help identify and localize both the tumor and textiloma [5-8,13-15].

MFH is an aggressive tumor with a high potential for metastasis to other parts of the body. The liver is the most commonly involved site of metastatic sarcomas, occurring in 64%–70% of patients [13-15]. The current treatment of choice for primary MFH is surgical resection. In order to improve survival in patients with MFH, in addition to complete resection of the primary tumor as well as isolated peritoneal or hepatic metastases where possible, an early multidisciplinary approach is also important [11-15]. Unfortunately, our patient had local recurrence of the tumor with liver metastases 14 months after the operation. Despite treatment, the patient died of progressive disease 2 months later.

## Conclusions

This case report shows that primary intra-abdominal MFH can present as a complication of long-lasting RSG. Therefore, clinicians must remember this while establishing the differential diagnosis for patients with a history of previous abdominal surgery and presenting with symptoms associated with both the tumor and systemic inflammatory response.

## Consent

Written informed consent was obtained from the patient for publication of this case report and accompanying images. A copy of the written consent is available for review from the Editor-in-Chief of this journal.

## Competing interests

The authors declare that they have no competing interests.

## Authors’ contributions

HII assisted the senior surgeon. MK performed the operation, designed the research, performed and analyzed the data, and wrote the paper. Both authors read and approved the final manuscript.
